# Altered mRNAs Profiles in the Testis of Patients With “Secondary Idiopathic Non-Obstructive Azoospermia”

**DOI:** 10.3389/fcell.2022.824596

**Published:** 2022-05-12

**Authors:** Dongdong Tang, Kuokuo Li, Mingrong Lv, Chuan Xu, Hao Geng, Chao Wang, Huiru Cheng, Xiaojin He, Yan Zhang, Yunxia Cao

**Affiliations:** ^1^ Reproductive Medicine Center, Department of Obstetrics and Gynecology, The First Affiliated Hospital of Anhui Medical University, Hefei, China; ^2^ NHC Key Laboratory of Study on Abnormal Gametes and Reproductive Tract (Anhui Medical University), Hefei, China; ^3^ Key Laboratory of Population Health Across Life Cycle (Anhui Medical University), Ministry of Education of the People’s Republic of China, Hefei, China; ^4^ Department of Clinical Laboratory, Renmin Hospital of Wuhan University, WuHan, China

**Keywords:** non-obstructive azoospermia, idiopathic, secondary, mRNA, profile

## Abstract

**Background:** Non-obstructive azoospermia (NOA) is the most severe form of male infertility. Currently, known causative factors, including congenital and several acquired causes only account for approximately 30% of NOA cases. The causes for NOA remain unclear for most patients, which is known as idiopathic (iNOA). However, whether iNOA is due to congenital defects or acquired abnormalities is a confusing problem due to the delayed diagnosis of this frustrating condition until the childbearing age. Therefore, we collected several cases with “secondary idiopathic NOA” and detected the altered mRNAs profiles in the testicular tissues to explore the possible molecular basis.

**Materials and Methods:** In this study, several patients with a previous history of natural pregnancy with their partners before, who were diagnosed as iNOA based on the outcomes of routine semen analysis and multiple testis biopsies now, were enrolled. Some known risk factors and genetic factors were excluded. Therefore, we defined this phenotype as “secondary idiopathic NOA.” To explore the possible molecular basis of this disease, we performed mRNA expression analysis through next-generation sequencing on three cases and other three patients with obstructive azoospermia as controls. Bioinformatics analyses were conducted to assess differentially expressed genes and possible biological mechanisms involved in the disease. Quantitative real-time reverse transcription polymerase chain reaction assays were applied to confirm the results in several selected mRNAs involved in stages and metabolism of Sertoli cells.

**Results:** A series of mRNAs were found to be altered in testicular tissues between patients with “secondary idiopathic NOA” and controls, including 6,028 downregulated and 3,402 upregulated mRNAs. Gene Ontology (GO) analysis and Kyoto Encyclopedia of Genes and Genome (KEGG) analyses revealed a range of GO and KEGG terms, such as cellular process involved in reproduction, protein degradation, and absorption.

**Conclusion:** The present study introduces a novel classification called “secondary idiopathic NOA.” We provide a global view of the altered mRNAs involved in spermatogenetic failure in these cases. Regarding the limited samples, further studies should be taken to understand this new classification.

## Introduction

As the most severe form of male infertility, non-obstructive azoospermia (NOA), is a devastating global health problem accounting for 10–15% of male infertility (Tuttelmann et al., 2011; Dabaja and Schlegel, 2013; Tournaye et al., 2017). Several known causative factors, including several congenital causes (e.g., chromosomal abnormalities, Y chromosome microdeletion, cryptorchidism, and monogenetic disease-causing mutations) and some acquired causes (e.g., radiotherapy/chemotherapy, orchitis, prior testicular torsion, and application of drugs inducing spermatogenic disorder), only account for approximately 30% of NOA cases. The causes for NOA in most patients remain unclear, which were also known as idiopathic NOA (iNOA) (Jungwirth et al., 2012; Practice Committee of the American Society for Reproductive Medicine, 2015; Wu et al., 2021). However, whether iNOA is due to congenital defects or acquired abnormalities is a confusing problem due to the delayed diagnosis of this frustrating condition until the childbearing age. In other words, we do not really understand whether the lack of sperm in testis in iNOA patients is a primary problem, or a secondary process. A few specialized cases diagnosed as iNOA who have a previous history of natural pregnancy have drawn our attention. Whether a series of internal or external environmental factors can lead to this serious fertile problem remains unknown.

Along with the development of microdissection testicular sperm extraction, some sperm can be retrieved from patients with iNOA and results in biological offspring through intracytoplasmic sperm injection (Caroppo et al., 2019; Ichioka et al., 2020; Li et al., 2020). Nonetheless, only approximately 10% of iNOA patients can benefit from these approaches, whereas no sperm can be found in the testis in the majority of iNOA cases (Dabaja and Schlegel, 2013). Therefore, the early identification, diagnostic assessment, and early intervention are very significant for patients with iNOA who once had sperm. Unnecessary surgical interventions and application of assisted reproductive techniques with a low success rate can be avoided. Fertility preservation with a simpler procedure and higher success rate of fertility can provide a better option for these patients. Accordingly, effective understanding and classification of iNOA is therefore indispensable.

To improve the understanding of “secondary iNOA” and characterization of transcriptomic profiles of testicular tissues in these cases, frozen testicular tissue samples from three patients with “secondary iNOA” and three patients with “obstructive azoospermia (OA)” used as controls were sent to perform mRNA expression profiling by next-generation sequencing (NGS). A series of altered mRNAs in testicular tissues were identified between these two groups. This study mainly introduces a novel classification of iNOA called “secondary iNOA” and provides a global view of the altered mRNAs of testicular tissues in these cases.

## Materials and Methods

### Study Design

**FIGURE 1:**
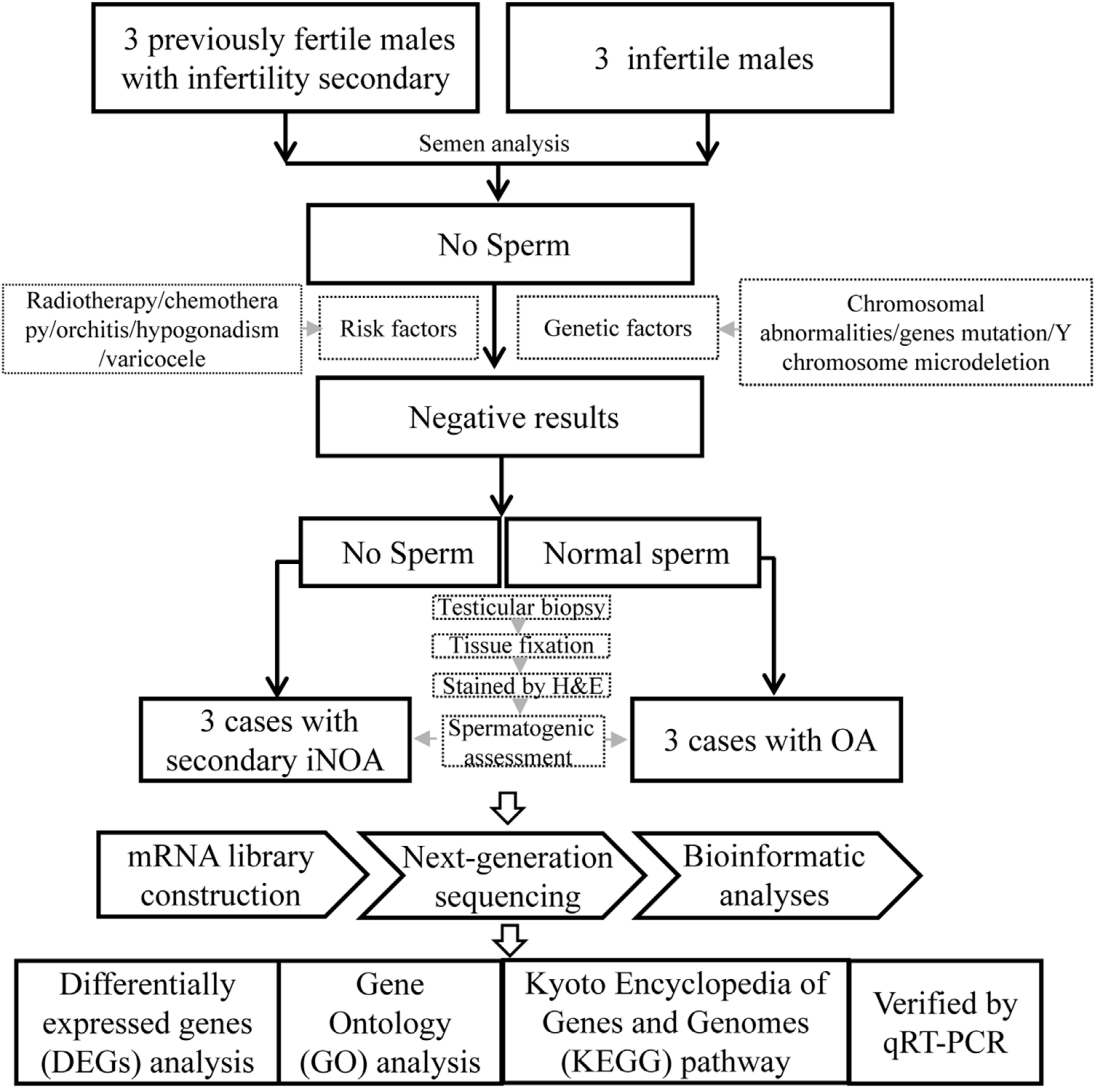
The flowchart of the experimental design.

Three patients with “secondary iNOA” and infertility were recruited as the case group, and three patients with OA as controls from the First Affiliated Hospital of Anhui Medical University. All the six patients underwent testicular biopsy. A small piece of testicular tissue was fixed and stained to assess the spermatogenic function. Another piece of tissue was frozen immediately and then *mRNA* sequencing was performed. Further bioinformatic analyses were performed to select differentially expressed genes (DEGs), and conduct Gene Ontology (GO) and Kyoto Encyclopedia of Genes and Genomes (KEGG) pathway enrichment analyses. The expression of some important DEGs selected were determined through quantitative reverse transcription polymerase chain reaction (qRT-PCR) to indicate the potential molecular basis of this disease. The experimental design flowchart of this study is summarized in Figure 1.

### Participants

Three patients with “secondary iNOA” were recruited as the case group, in which cases had to meet the following criteria: 1) at least two semen analysis after centrifugation suggest azoospermia; 2) without the risk factors, including radiotherapy, chemotherapy, orchitis, hypogonadism, varicocele, and genetic factors, such as chromosomal abnormalities, Y chromosome microdeletion, and gene mutations 3) with normal spermatogenic function or history of natural pregnancy before, however, without sperm in testis by testicular biopsy. The flowchart for the three patients with OA (control) was the same as the one previously described, except for (3) that, in this case, corresponded to normal spermatogenic function and with normal sperm in testis by testicular biopsy.

### Ethical Approval

All participants signed a written informed consent to participate in the study. The study was approved by the review board committee of the First Affiliated Hospital of Anhui Medical University (20160106, 1 March 2016), and it was conducted in accordance with the Declaration of Helsinki.

### Specimen Collection and Hematoxylin and Eosin Staining

Testicular biopsy was conducted to assess the spermatogenic function in the testis of the six patients. A small piece of testicular tissue was fixed in Bouin’s solution, and subsequent H&E staining was performed according to routine protocols to assess the histopathological features of testicular tissues as previously described (Zuo et al., 2017; Tang et al., 2021). In brief, the fixed testicular tissues were embedded in paraffin and 5-µm thick sections were dewaxed, rehydrated, and stained with H&E subsequently. Another piece of testicular tissue was frozen at −80°C in a cryopreservation tube with RNA later tissue storage reagent immediately after dissection.

### Construction of the mRNA Library, Next-Generation Sequencing, and Bioinformatic Analyses

TRIzol (Life Technologies, Carlsbad, CA, United States) was used to extract total RNA from the six testicular tissues, respectively. Subsequently, the NEBNext^®^ Multi-plex mRNA Library Prep Set (Illumina^®^) was used to construct a mRNA library. The sequencing analysis was performed on a Hiseq X (Illumina) using the HiSeq X Reagent Kit v2. FastQC and the fastp software were used to estimate the quality of the sequencing and removed primers with low quality (quality below 15 and length of reads below 40 bp). HISAT2 was used for mapping sequencing to human reference genome and estimate the fraction of sequencing mapping to exon, intron, and intergenic sequences.

### Quantitative Realtime PCR (QRT-PCR) Analysis

In order to confirm the data generated from sequencing efforts were correct, QRT-PCR validation of specific mRNA was performed. Total RNA was extracted from the six testicular tissues by TRIzol (Life Technologies, Carlsbad, CA, United States) previously described and transcribed into cDNA using the PrimeScript RT Reagent Kit (Takara, Shiga, Japan) according to the manufacturer’s protocol. The obtained cDNAs were used as templates for subsequent quantitative real-time PCR conducted using the Light Cycler 480 SYBR Green I Master (Roche, Switzerland, Germany). The expression of mRNAs was quantified according to the 2-ΔΔCt method. β-actin was used as an internal control. The primers used were listed in Supplementary Table S1.

### Statistical Analyses

StringTie was used to quantify gene expression (Pertea et al., 2015). The Deseq2 was used to perform differential expression analysis between NOA cases and unaffected individuals using the Wilcox rank sum test method (Love et al., 2014). A statistical *p* value <0.05 and |log2 (fold change)| > 1 were defined as significant differential expression. The R prcomp function was used to perform principal component analysis (PCA) to cluster samples based on gene expression. In addition, we performed gene ontology (GO) and Kyoto Encyclopedia of Genes and Genomes (KEGG) pathway enrichment analysis for DEGs between cases and controls using Fisher’s exact test based tools clusterProfiler and ToppGene (Chen et al., 2009; Yu et al., 2012). The *p* value was adjusted using the Benjamin–Hochberg method and the adjusted *p* < 0.05 was considered significant enrichment. The rMATS was used to perform alternative splicing analysis and *p* < 0.05 was defined as significantly alternative splicing. QRT-PCR results are presented as mean ± standard deviation (SD) of triplicates. Student’s t-tests were used for comparing mRNA levels between two groups, with *p* < 0.05 as the threshold of significance.

## Results

### Clinical Features of the Three Patients With Secondary iNOA

The age of the three patients (named as case1, case2, and case3) was 38, 28, and 33 years at the time of diagnosis, respectively. The three cases presented self-reported normal or nearly normal results of semen analysis, and had a history of natural pregnancies with their partners before, which indicated prior normal fertility. Cases 2 had his own first child and the partners of cases 1 and 3 had a history of induced abortions. The medical history indicated no prior history of surgery or no reproduction-related diseases. Preoperative examinations presented normal somatic karyotypes and normal results of the Y-chromosome microdeletions. Abnormally increased levels of follicle stimulating hormone were detected in Case 1 and Case 2 and normal testosterone were detected in all the three cases. The detailed clinical features of these three cases are showed in Table 1.

**TABLE 1:** Clinical features of cases with secondary iNOA and controls.

Individual	Case 1	Case 2	Case 3	Control 1	Control 2	Control 3
Age	38	28	33	24	26	29
BMI	25.22	22.31	27.09	28.85	26.32	21.62
Secondary sexual characteristics	Normal	Normal	Normal	Normal	Normal	Normal
Testicular volume (Left/Right, ml)	10/10	12/12	10/10	15/15	12/12	12/12
Somatic karyotype	46,XY	46,XY	46,XY	46,XY	46,XY	46,XY
Y Chromosome microdeletions	No	No	No	No	No	No
Sex hormone						
Follicle-stimulating hormone (IU/L)	37.21	16.22	8.15	1.58	2.28	7.41
Luteinizing hormone (IU/L)	9.20	4.42	2.57	9.68	1.13	5.27
Testosterone (nmol/L)	22.01	9.10	11.36	11.22	23.47	11.78
Prolactin (ng/ml)	9.36	14.33	15.10	—	20.03	12.26
Estradiol (pmol/L)	178	100	44	—	118	148
History of previous pregnancies	Induced abortion	Having a child	Induced abortion	No	No	No
Characteristics of lifestyle						
Exposure to environmental toxins	No	No	No	No	No	No
Exposure to high temperatures	No	No	No	No	No	No
Exposure to radiation or cytotoxic agents	No	No	No	No	No	No
Physical activity	Yes	Yes	Yes	Yes	Yes	Yes
Smoking	No	Yes	No	Yes	Yes	No
Alcohol consumption	Occasional	Occasional	Occasional	Occasional	Occasional	Occasional

### Altered mRNAs Expression Profiles in Testicular Tissues Between Patients With Secondary iNOA and Controls

**FIGURE 2:**
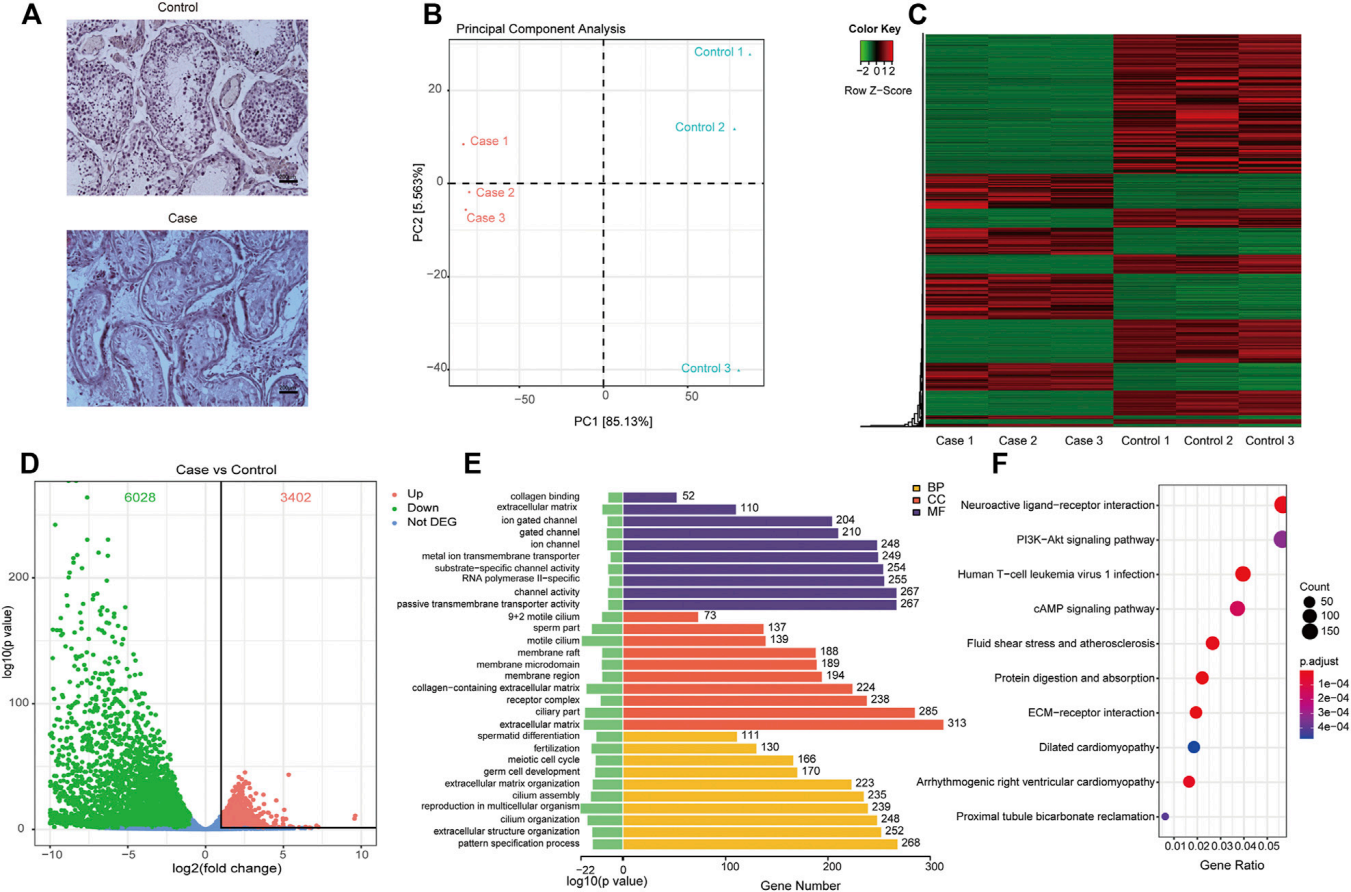
Differentially expressed genes and their related functions. **(A)** H&E staining of testicular tissue; **(B)** principal component analysis of expressed genes distinguish cases and controls; **(C)** heatmap and phylogenetic tree of differentially expressed genes; **(D)** the differentially expressed genes. Red dots indicate significantly upregulated genes in cases. Green dots indicate significantly downregulated genes in cases and that the genes were not significantly differentially expressed in cases were indicated by blue dots; **(E)** gene ontology functional enrichment of significantly differentially expressed genes; **(F)**, KEGG functional enrichment of significantly differentially expressed genes.

As shown in Figure 2A, the absence of germ cells was observed in the testicular tissues of patients with secondary iNOA, compared to that of the normal control. To explore the potential molecular basis of this phenotype, we performed RNA-seq of six testicular tissues to decode the gene expression patterns in secondary iNOA cases and controls. The quality of RNA-seq was satisfied based on the quality value, mapping distribution, and FPKM density (Supplementary Figure 1, Supplementary Tables S2, S3). We performed PCA analysis based on expressed genes in the two groups. Three secondary iNOA cases clustered together, which indicated the functional similarity. For the controls, we found a relatively high distance of control 3 compared to that of controls 1 and 2, which might indicate heterogeneity of testicular tissue (Figure 2B). Although slight differences among controls, the two different phenotype groups show clear boundaries, which indicate their suitability for further analysis (Figure 2B).

### Bioinformatics Analyses of Differentially Expressed Genes in Testicular Tissues in Patients With Secondary iNOA and Controls

Based on bioinformatics analyses, a range of differentially expressed mRNAs were found in testicular tissues in patients with secondary iNOA and controls, including 6,028 downregulated and 3,402 upregulated mRNAs, which allowed us to generate an expression heatmap (Figure 2C, Supplementary Table S4). In this heatmap, the rows represent mRNAs and columns represent samples, with cluster analyses grouping samples based on their similar expression profiles. This successful hierarchical clustering supported the reliability of the mRNA sequencing data.

A scatter plot of sample log_2_-fold changes was generated, offering a visualization of differential mRNA expression (Figure 2D). In this plot, the central blue plot indicates no difference, while those mRNAs above the upper (red) and below the lower plots (green) were at least 2-fold changed between the secondary iNOA cases and controls.

To estimate the functional difference between cases and controls, we performed GO enrichment and KEGG pathway analysis (Supplementary Tables S5, S6). We found that the most significant enrichment cellular component and biological process terms were related to reproduction biology, whereas the molecular function terms were related to the channel activity. The 10 most enriched GO terms for the three GO categories (cellular component, biological process, and molecular function) are presented in Table 2 and Figure 2E, including the cellular process involved in reproduction, biological process domain, extracellular matrix, cellular component domain, extracellular matrix structural constituent, and molecular function domain. For the KEGG pathway, we found that genes were enriched in protein digestion and absorption, cAMP signaling pathway, and PI3K-Akt signaling pathway. The top 30 most enriched KEGG pathways are presented in Table 3 and Figure 2F. To decode more detailed functional differences between cases and controls, we selected the 30 most significantly downregulated (Table 4) and the 30 most significantly upregulated genes (Table 5). For the DEGs, the downregulated genes in patients show a higher significance in fold change expression and statistical value, compared to upregulated genes. These results were consistent with the phenotype of testicular tissue carrying only Sertoli cells in patients with secondary iNOA. We also performed functional analysis for these two kinds of gene groups (Supplementary Tables S7, S8). The top 30 most significant downregulated genes were enriched in reproduction-related functions, which was consistent with 6,028 downregulated functional enrichments (Supplementary Tables S5, S7). However, the top 30 most significantly upregulated genes were enriched in secretion by cell, positive regulation of receptor recycling, leukocyte mediated immunity, lysosome, and cytoplasmic vesicle membrane, which indicate the potential functional differences between cases and controls.

**TABLE 2:** Top 10 most enriched gene ontology terms.

GO ID	Terms	Description	Gene Ratio	P	Adjust P
GO:0022412	BP	Cellular process involved in reproduction	239/7,844	5.85E-23	3.72E-19
GO:0044782	BP	Cilium organization	248/7,844	3.05E-19	9.7E-16
GO:0060271	BP	Cilium assembly	235/7,844	1.96E-17	4.14E-14
GO:0009566	BP	Fertilization	130/7,844	4.17E-17	6.63E-14
GO:0043062	BP	Extracellular structure organization	252/7,844	1.12E-16	1.42E-13
GO:0030198	BP	Extracellular matrix organization	223/7,844	1.81E-16	1.76E-13
GO:0007389	BP	Pattern specification process	268/7,844	1.94E-16	1.76E-13
GO:0007281	BP	Germ cell development	170/7,844	3.38E-15	2.69E-12
GO:0051321	BP	Meiotic cell cycle	166/7,844	7.14E-15	5.05E-12
GO:0048515	BP	Spermatid differentiation	111/7,844	1.6E-14	1.02E-11
GO:0031514	CC	Motile cilium	139/8,443	3.01E-22	2.31E-19
GO:0031012	CC	Extracellular matrix	313/8,443	3.12E-21	1.2E-18
GO:0044441	CC	Ciliary part	285/8,443	1.62E-20	4.14E-18
GO:0062023	CC	Collagen-containing extracellular matrix	224/8,443	9.25E-20	1.78E-17
GO:0097223	CC	Sperm part	137/8,443	6.58E-17	1.01E-14
GO:0043235	CC	Receptor complex	238/8,443	3.14E-12	4.02E-10
GO:0098857	CC	Membrane microdomain	189/8,443	1.31E-11	1.43E-09
GO:0097729	CC	9 + 2 motile cilium	73/8,443	1.65E-11	1.58E-09
GO:0045121	CC	Membrane raft	188/8,443	1.91E-11	1.63E-09
GO:0098589	CC	Membrane region	194/8,443	2.32E-11	1.78E-09
GO:0005201	MF	Extracellular matrix structural constituent	110/7,872	2.54E-11	3.04E-08
GO:0022836	MF	Gated channel activity	210/7,872	5.71E-09	3.42E-06
GO:0005216	MF	Ion channel activity	248/7,872	1.17E-08	3.95E-06
GO:0022839	MF	Ion gated channel activity	204/7,872	1.32E-08	3.95E-06
GO:0015267	MF	Channel activity	267/7,872	2.48E-08	5.44E-06
GO:0022838	MF	Substrate-specific channel activity	254/7,872	2.92E-08	5.44E-06
GO:0022803	MF	Passive transmembrane transporter activity	267/7,872	3.24E-08	5.44E-06
GO:0005518	MF	Collagen binding	52/7,872	3.63E-08	5.44E-06
GO:0001228	MF	DNA-binding transcription activator activity, RNA polymerase II-specific	255/7,872	1.12E-07	1.49E-05
GO:0046873	MF	Metal ion transmembrane transporter activity	249/7,872	6.38E-07	7.36E-05

**TABLE 3:** Top 30 most enriched KEGG pathway.

Pathway ID	Description	GeneRatio	*p*	Adjust P
hsa04974	Protein digestion and absorption	73/3,400	2.68E-09	7.85E-07
hsa04512	ECM-receptor interaction	64/3,400	4.85E-09	7.85E-07
hsa05166	Human T-cell leukemia virus 1 infection	132/3,400	3.16E-08	3.41E-06
hsa04080	Neuroactive ligand-receptor interaction	190/3,400	1.63E-07	1.32E-05
hsa05418	Fluid shear stress and atherosclerosis	88/3,400	3.09E-07	2.00E-05
hsa05412	Arrhythmogenic right ventricular cardiomyopathy	54/3,400	5.53E-07	2.98E-05
hsa04024	cAMP signaling pathway	124/3,400	3.21E-06	1.48E-04
hsa04151	PI3K-Akt signaling pathway	189/3,400	8.06E-06	3.27E-04
hsa04964	Proximal tubule bicarbonate reclamation	20/3,400	1.14E-05	4.11E-04
hsa05414	Dilated cardiomyopathy	61/3,400	1.69E-05	4.76E-04
hsa04510	Focal adhesion	114/3,400	1.70E-05	4.76E-04
hsa04925	Aldosterone synthesis and secretion	62/3,400	1.76E-05	4.76E-04
hsa04514	Cell adhesion molecules	88/3,400	1.93E-05	4.82E-04
hsa04020	Calcium signaling pathway	113/3,400	3.15E-05	7.29E-04
hsa05410	Hypertrophic cardiomyopathy	57/3,400	3.65E-05	7.88E-04
hsa04218	Cellular senescence	90/3,400	5.44E-05	1.05E-03
hsa04261	Adrenergic signaling in cardiomyocytes	87/3,400	5.52E-05	1.05E-03
hsa04022	cGMP-PKG signaling pathway	95/3,400	7.20E-05	1.30E-03
hsa00010	Glycolysis/gluconeogenesis	44/3,400	7.98E-05	1.36E-03
hsa05165	Human papillomavirus infection	173/3,400	9.12E-05	1.48E-03
hsa05225	Hepatocellular carcinoma	95/3,400	9.79E-05	1.51E-03
hsa04926	Relaxin signaling pathway	75/3,400	1.59E-04	2.34E-03
hsa04145	Phagosome	86/3,400	2.01E-04	2.66E-03
hsa04658	Th1 and Th2 cell differentiation	56/3,400	2.05E-04	2.66E-03
hsa05222	Small cell lung cancer	56/3,400	2.05E-04	2.66E-03
hsa04972	Pancreatic secretion	61/3,400	2.19E-04	2.73E-03
hsa05169	Epstein-Barr virus infection	110/3,400	2.30E-04	2.76E-03
hsa04610	Complement and coagulation cascades	52/3,400	2.87E-04	3.32E-03
hsa04970	Salivary secretion	56/3,400	3.06E-04	3.42E-03
hsa04725	Cholinergic synapse	66/3,400	3.22E-04	3.47E-03

**TABLE 4:** Top 30 most significantly downregulated genes in patients.

Gene	Case	Control	log2FoldChange	*p* value	Adjust *p*
*TSSK6*	105.68	47,491.96	−8.81	0	0
*ANKRD7*	399.76	128,144.62	−8.32	0	0
*TUBA3D*	242.19	47,463.79	−7.61	1.18E-264	7.39E-261
*YBX2*	131.35	107,165.69	−9.67	5.62E-243	2.64E-239
*ACTRT3*	159.28	12,314.70	−6.27	3.62E-231	1.36E-227
*ACRBP*	271.71	52,922.49	−7.60	4.47E-231	1.40E-227
*CRISP2*	36.55	77,553.97	−11.03	3.95E-226	1.06E-222
*WDR62*	239.12	28,238.56	−6.88	9.41E-221	2.21E-217
*ZMYND10*	175.00	53,558.13	−8.25	7.10E-219	1.48E-215
*SPTBN2*	343.63	26,742.28	−6.28	2.47E-218	4.65E-215
*FAM186B*	40.21	14,508.79	−8.50	1.99E-216	3.40E-213
*CATSPERZ*	78.08	80,993.20	−10.02	3.13E-213	4.91E-210
*CFAP43*	36.09	12,876.27	−8.49	7.33E-213	1.06E-209
*C16orf71*	52.84	13,058.29	−7.94	1.95E-208	2.61E-205
*TLCD3B*	26.74	11,208.07	−8.74	5.95E-205	7.46E-202
*CCDC136*	190.80	84,940.90	−8.80	4.87E-201	5.72E-198
*TMEM225B*	214.06	17,419.52	−6.35	1.47E-198	1.63E-195
*C15orf48*	77.24	9,628.02	−6.97	4.25E-188	4.44E-185
*CDC20*	72.18	10,689.50	−7.20	5.60E-187	5.54E-184
*GAPDHS*	96.13	46,612.74	−8.93	6.36E-187	5.98E-184
*IQCD*	64.30	9,816.27	−7.25	9.93E-184	8.89E-181
*CCDC54*	64.32	14,981.57	−7.87	5.55E-180	4.74E-177
*KLHL10*	23.14	10,851.46	−8.88	1.17E-178	9.60E-176
*TSNAXIP1*	271.40	9,708.68	−5.16	2.37E-177	1.86E-174
*SPATA24*	100.71	10,108.98	−6.64	2.68E-177	2.01E-174
*DDX4*	6.81	23,792.88	−11.74	1.35E-176	9.74E-174
*PROCA1*	332.44	26,495.39	−6.32	9.20E-175	6.41E-172
*PKMYT1*	93.81	10,287.66	−6.79	1.89E-174	1.27E-171
*CCDC168*	21.70	5,875.62	−8.06	5.84E-173	3.79E-170
*CCDC96*	319.08	18,734.81	−5.87	2.39E-172	1.50E-169

**TABLE 5:** Top 30 most significantly upregulated genes in patients.

Gene	Case	Control	log2FoldChange	*p* value	Adjust P
*B2M*	87,741.46	15,138.13	2.53	4.60E-46	1.44E-44
*U2AF1*	1,209.73	30.26	5.34	3.18E-44	9.44E-43
*HEXB*	9,323.97	2,120.95	2.14	1.85E-42	5.09E-41
*NGFR*	5,076.68	1,015.99	2.32	1.11E-40	2.89E-39
*ARHGDIB*	3,512.76	590.82	2.57	1.37E-38	3.30E-37
*CTSC*	13,078.00	1,902.51	2.78	2.40E-37	5.48E-36
*DAB2*	5,884.26	1,068.64	2.46	4.64E-37	1.05E-35
*C1S*	37,879.53	6,484.56	2.55	4.66E-37	1.05E-35
*MGP*	16,324.08	3,732.20	2.13	6.30E-37	1.41E-35
*VIM*	189,053.12	40,274.34	2.23	3.74E-35	7.75E-34
*EID1*	30,949.22	8,249.67	1.91	1.42E-34	2.85E-33
*CPE*	58,219.52	10,913.72	2.42	4.32E-34	8.52E-33
*TPM4*	27,431.16	6,587.87	2.06	9.78E-34	1.90E-32
*NSG1*	18,918.34	2,639.12	2.84	1.35E-33	2.62E-32
*GSTA4*	12,493.00	3,139.39	1.99	1.37E-33	2.66E-32
*ANO6*	6,258.16	1,626.75	1.94	3.96E-32	7.32E-31
*ARMCX3*	7,699.53	1,653.00	2.22	6.08E-32	1.12E-30
*FOSL2*	2,749.84	626.82	2.13	1.16E-31	2.11E-30
*CMTM6*	15,488.52	4,646.03	1.74	2.35E-31	4.22E-30
*PTCH2*	18,472.23	3,941.54	2.23	4.20E-31	7.46E-30
*TCEAL9*	15,877.11	4,364.94	1.86	4.65E-31	8.21E-30
*AMIGO2*	6,881.53	1,345.08	2.36	8.09E-31	1.41E-29
*COL27A1*	10,573.52	1,119.48	3.24	2.19E-29	3.58E-28
*VCAN*	4,228.06	814.11	2.38	1.63E-28	2.56E-27
*APOL2*	3,186.35	734.73	2.12	2.24E-28	3.50E-27
*CMYA5*	2,612.57	198.58	3.72	2.70E-28	4.21E-27
*ARMCX1*	2,717.68	598.99	2.18	4.62E-28	7.13E-27
*ACSL4*	11,956.89	3,362.75	1.83	1.33E-27	2.02E-26
*CFI*	1,003.74	135.95	2.88	5.71E-27	8.43E-26
*PLXDC2*	8,216.64	1,897.98	2.11	5.82E-27	8.59E-26

The hard filtering DEG threshold between cases and controls can result in random errors in functional enrichment. To better decode the functional differences of expressed genes between cases and controls, we performed GSEA functional analysis (Supplementary Table S9). We found that the spermatogenesis-related functions were also downregulated in the cases, which was consistent with DEGs related to function (Supplementary Tables S5, S7).

In addition, we performed alternative splicing analysis for five aspects including alternative 3′ splicing site, alternative 5′ splicing site, mutually exclusive exons, retained intron, and skipped exon, and found 346, 358, 583, 110, and 3,135 significant alternative splicing, respectively (Supplementary Table S10).

### Validation of mRNAs Expression Through qRT-PCR

**FIGURE 3:**
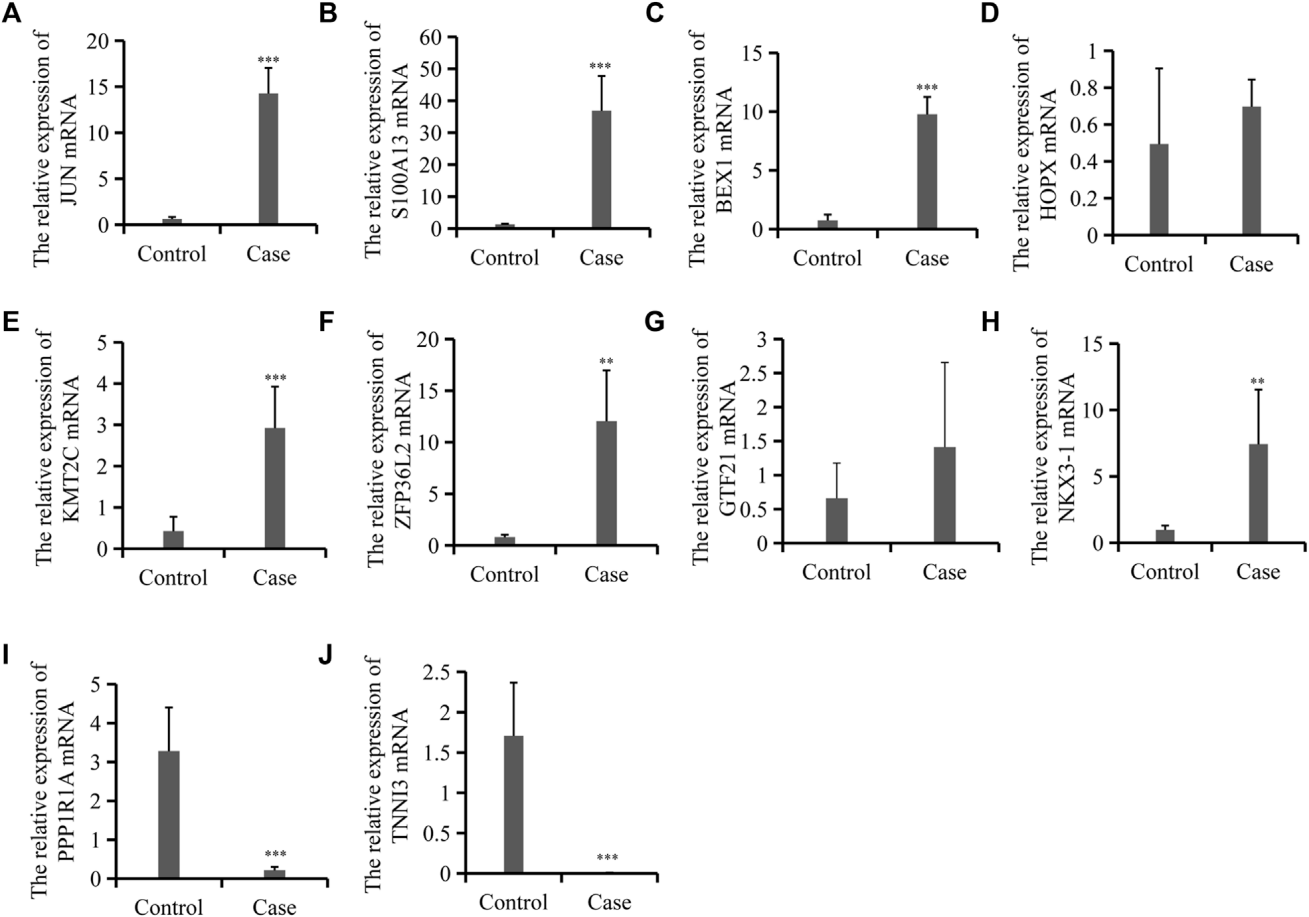
Expression validation for 10 differentially expressed mRNAs between the control and cases group. **(A–J)**. Relative mRNA levels of JUN, S100A13, and BEX1 et al. as indicated by qRT-PCR. Data are mean ± SD (*n* = 3); *p < 0.05, **p < 0.01, ***p < 0.001.

Regarding the common cell type shared by the two groups and the important role of Sertoli cells, of the 9,430 differentially expressed mRNAs, we selected eight upregulated and two downregulated mRNAs involved in the important function of Sertoli cells for further investigation. Four out of eight upregulated mRNAs were DEGs in Sertoli cells in different ages (a, b, and c), and the others were metabolism-related genes and the top candidate gene expression regulators. We further used qRT-PCR to validate the expression of these selected mRNAs and the results confirmed the expression detected during mRNA sequencing. As shown in Figure 3, the expressions of JUN, the marker of Stage_a Sertoli cell, and S100A13/BEX1, the markers of Stage_b Sertoli cell, were increased significantly. Moreover, ZFP36L2 and KMT2C, which might be the master regulators or transcription factors in Stage_b Sertoli cells, were also upregulated in the cases group. However, the expression of HOPX, the top candidate gene expression regulator in Stage_c Sertoli cells, was of no significant difference compared with the control. In addition, the cardiac differentiation gene TNNI3 and the energy metabolism-related gene PPP1R1A were decreased dramatically in the case group, which was consistent with the RNA sequencing results. These findings indicated that secondary iNOA testicular tissues were mainly composed of immature Sertoli cells (including Stage_a and Stage_b Sertoli cells).

## Discussion

The treatment for iNOA is a Gordian knot. Considering the occurrence of secondary iNOA, a deeper understanding of these special cases is gaining importance. To the best of our knowledge, because of the extreme difficulties in secondary iNOA identification, little attention has been paid to this condition. This study describes a novel type of iNOA called secondary iNOA for the first time and shows the altered mRNA profiles in the testicular tissues of patients with this condition.

The definition of secondary iNOA is ostensibly straightforward. According to the analogies used for other secondary diseases, a person with secondary iNOA is someone who presented sperm in the testis tissues before, but currently suffers from NOA without any known related etiologies. Nevertheless, several factors prevent the identification and understanding of these secondary iNOA cases. First, most of the patients with iNOA were not diagnosed until the childbearing age. Therefore, we do not have information related to their fertile function before. In other words, we cannot identify whether iNOA is a primary situation or a secondary disorder. Second, although clinicians can encounter occasionally cases of iNOA whose partners had a natural pregnancy before, the first issue requiring consideration is the biological parent–offspring relation. However, it is an ethically questionable approach to investigate the parent–offspring relationship. Therefore, we enrolled only three patients with secondary iNOA in nearly 5 years due to the strict inclusion criteria. The three patients not only had a history of natural pregnancies, but also had self-reported normal or nearly normal results of semen analysis before.

Somatic and germ cells collectively constitute adult testicular tissues and act synergistically in spermatogenesis. It is beyond doubt that germ cells play indispensable roles in the process of spermatogenesis (Chen et al., 2018; Wang et al., 2018). In fact, Sertoli cells, which are located around and are in direct contact with germ cells, function as scaffolds and “nurse” cells in the spermatogenic microenvironment (Zhao et al., 2020). Therefore, further research into Sertoli cells may help understand the defects of the somatic microenvironment in iNOA. Moreover, some studies have reported the association between the testicular somatic microenvironment and male infertility (Ma et al., 2013; Zhao et al., 2020). Ma et al. (2013) found that ultrastructural features of Sertoli cells from testicular tissues in NOA patients were abnormal and the expression levels of some genes decreased. With the development of single-cell RNA sequencing analysis, intensive and high resolutive investigation of different cell populations and function analysis of diverse cells in testicular tissues are becoming a reality. Zhao et al. (2020) found the central role of Sertoli cells in the testicular somatic microenvironment and severely impaired Sertoli cells in testicular tissues from iNOA patients by single-cell RNA sequencing analysis. In our study, although only routine mRNA sequencing by NGS in testicular tissues was used, we still obtained several meaningful findings. Through bioinformatics analyses of GO enrichment and KEGG pathway analysis, a series of reproduction-related terms were enriched, such as cellular process involved in reproduction, fertilization, extracellular structure organization in biological process domain, extracellular matrix, collagen-containing extracellular matrix, receptor complex, membrane microdomain in cellular component domain, and ion channel activity in the molecular function domain. Similar results of bioinformatics analysis were found by Zhang et al. (2020), who conducted research in the microRNA profiles of patients with NOA. Some terms enriched in the bioinformatics analyses may be accounted for the different cell populations in the two groups. For example, cilium organization, cilium assembly, germ cell development, and sperm part were enriched due to the lack of germ cells and sperm in the case group.

Furthermore, we selected 10 DEGs to verify the reliability of the mRNA sequence. Eight out of 10 mRNAs were consistent with the NGS results. It was found that four genes differentially expressed in Sertoli cells in different stages (Stage_a, Stage_b, and Stage_c) indicated by Zhao et al. (2020), and four genes involved in Sertoli cell metabolism. The significantly increased expressions of JUN (marker of Stage_a Sertoli cell), and S100A13/BEX1 (markers of Stage_b Sertoli cell) indicated that secondary iNOA testicular tissues were mainly composed of immature Sertoli cells (including Stage_a and Stage_b Sertoli cells). These findings suggested that the changes of the somatic microenvironment patients with secondary iNOA are mainly manifested in Sertoli cells, which was consistent with the results of Zhao et al. (2020). Compared with similar studies, the background of the three patients in our study were relatively clear that they were all previously fertile males with secondary infertility. This prompted us to investigate the probable process of infertility caused by changes of Sertoli cells and to provide fertility preservation that could potentially serve as therapeutic targets for secondary iNOA treatment.

Although the findings of this study are interesting, they have some limitations. Particularly because of the factors mentioned above, the number of samples in the study is relatively small. Therefore, larger number of patients is needed to give definitive answers in our future work. Second, despite the single-cell RNA sequencing analysis having great advantages to identify different cell populations and differential mRNAs expressions in diverse cells in testicular tissues (Li et al., 2017; Shami et al., 2020; Guo et al., 2021; Mahyari et al., 2021), giving the limited conditions to obtain the patients’ testicular tissues again, the routine mRNA sequencing using the frozen testicular samples was conducted in this study instead of the single-cell RNA sequencing analysis which must use the fresh samples. Third, the cellular experiment was necessary to further verify these results, whereas it is hard to perform this experiment due to limited testicular tissue of patients.

## Conclusion

In summary, this present study introduces a novel classification of iNOA- the “secondary idiopathic NOA”, which should be given enough attention to provide fertility preservation for patients with this condition. Additionally, we provide a global view of the altered mRNAs in testicular tissues of patients with this disease. However, the differential mRNAs expression in diverse cell populations of testicular tissues remains unclear. Therefore, determining the specified genes and regulatory networks in the special cells is essential in future investigations.

## Data Availability

The datasets presented in this study can be found in online repositories. The names of the repository/repositories and accession number(s) can be found below: NCBI (accession: GSE190752).
