# Supplementary material for: Altered mRNAs Profiles in the Testis of Patients With “Secondary Idiopathic Non-Obstructive Azoospermia”
Source: Front Cell Dev Biol. 2022 May 12;10:824596. doi: 10.3389/fcell.2022.824596 (PMC9133692; doi:10.3389/fcell.2022.824596)
Supplement: Supplementary file 3 [file Table1.DOCX]

**Supplementary table 1. Primers Used for RT-qPCR Assays**

| **Primer Names** | **Primer Sequences (5'-3')** | **Annealing temperature (°C)** |
| --- | --- | --- |
| JUN-F | TCCAAGTGCCGAAAAAGGAAG | 60 |
| JUN-R | CGAGTTCTGAGCTTTCAAGGT | 60 |
| S100A13-F | GATAGCCTCAGCGTCAACGAG | 60 |
| S100A13-R | CCTGATTCACATCCAAGCTCTT | 60 |
| BEX1-F | GCAGTAAACAGTCTCAGCATGG | 60 |
| BEX1-R | GGCTCCCCTTTATTAGCAACTT | 60 |
| HOPX-F | GAGACCCAGGGTAGTGATTTGA | 60 |
| HOPX-R | AAAAGTAATCGAAAGCCAAGCAC | 60 |
| NKX3-1-F | CCCACACTCAGGTGATCGAG | 60 |
| NKX3-1-R | GAGCTGCTTTCGCTTAGTCTT | 60 |
| GTF2I-F | TTGTCGTCGGAACTGAAAGAG | 60 |
| GTF2I-R | CGATTTGCCTGGGTTGTAGAT | 60 |
| ZFP36L2-F | GAGAACAAATTCCGGGACCG | 60 |
| ZFP36L2-R | GCGTGGAGTTGATCTGGGAG | 60 |
| KMT2C-F | CTCGCAAAGATGGCGCTTC | 60 |
| KMT2C-R | TCTGTTTCCACAATCGTTTCTGT | 60 |
| PPP1R1A-F | CCACGGCAACGGAAGAAGAT | 60 |
| PPP1R1A-R | CCCCAGGTGATGTTCAACCA | 60 |
| TNNI3-F | TTTGACCTTCGAGGCAAGTTT | 60 |
| TNNI3-R | CCCGGTTTTCCTTCTCGGTG | 60 |
| β-actin-F | TGGCACCCAGCACAATGAA | 60 |
| β-actin-R | CTAAGTCATAGTCCGCCTAGAAGCA | 60 |
