# Supplementary figures and images for: Altered mRNAs Profiles in the Testis of Patients With “Secondary Idiopathic Non-Obstructive Azoospermia”
Source: Front Cell Dev Biol. 2022 May 12;10:824596. doi: 10.3389/fcell.2022.824596 (PMC9133692; doi:10.3389/fcell.2022.824596)

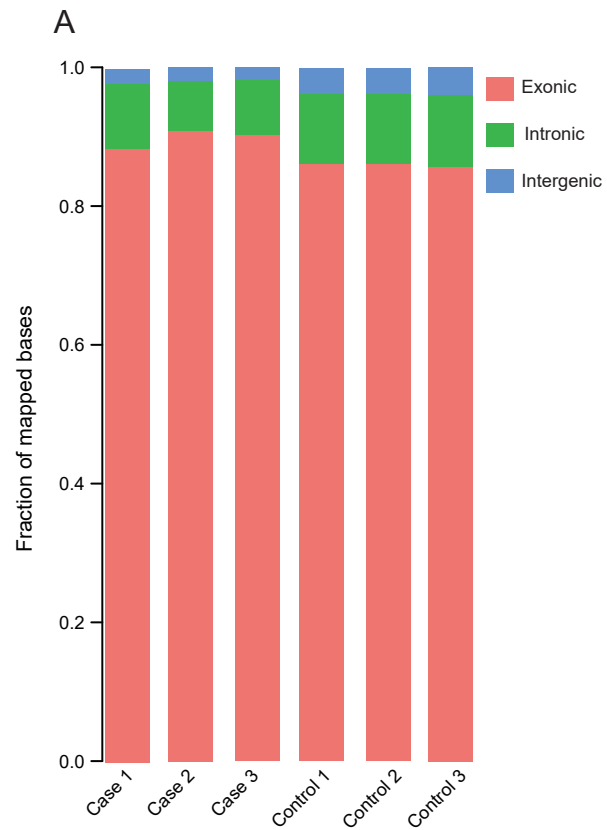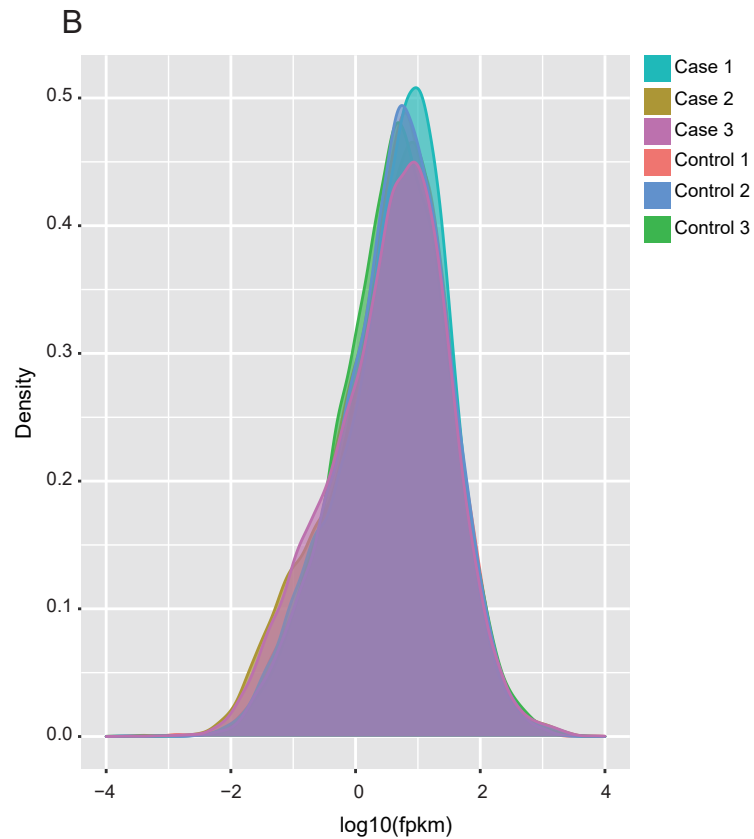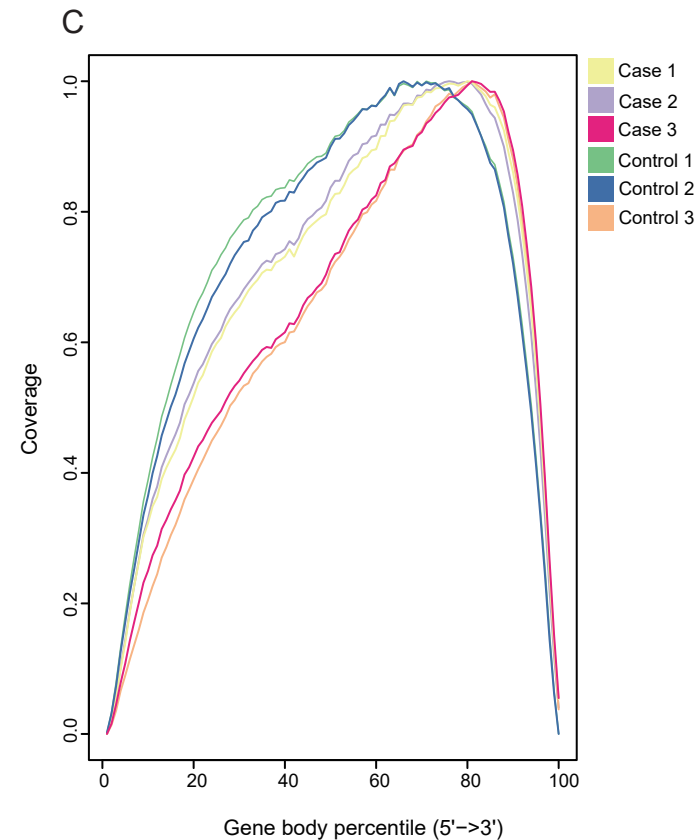

Supplement: Supplementary file 11 [file Image1.pdf]
